# Single-Cell Transcriptomics of Endothelial Cells in Upper and Lower Human Esophageal Squamous Cell Carcinoma

**DOI:** 10.3390/curroncol29100607

**Published:** 2022-10-14

**Authors:** Yongqiang Sha, Huhai Hong, Wenjie Cai, Tao Sun

**Affiliations:** 1 Center for Precision Medicine, School of Medicine and School of Biomedical Sciences, Huaqiao University, Xiamen 361021, China; 2 Departments of Radiation Oncology, First Hospital of Quanzhou Affiliated to Fujian Medical University, Quanzhou 362000, China

**Keywords:** esophageal squamous cell carcinoma, single cell RNA sequencing, endothelial cells, anti-angiogenesis, cell metastasis

## Abstract

Esophageal squamous cell carcinoma (ESCC) is a type of progressive and distant metastatic tumor. Targeting anti-angiogenic genes could effectively hinder ESCC development and metastasis, whereas ESCC locating on the upper or the lower esophagus showed different response to the same clinical treatment, suggesting ESCC location should be taken into account when exploring new therapeutic targets. In the current study, to find novel anti-angiogenic therapeutic targets, we identified endothelial cell subsets in upper and lower human ESCC using single-cell RNA sequencing (scRNA-seq), screened differentially expressed genes (DEGs), and performed gene ontology (GO) and Kyoto Encyclopedia of Genes and Genomes (KEGG) analysis. The results showed that common DEGs shared in the upper and the lower endothelial cells mainly are involved in vessel development, angiogenesis, and cell motility of endothelial cells by regulating PI3K-AKT, Rap1, Ras, TGF-beta, and Apelin signaling pathways. The critical regulatory genes were identified as *ITGB1*, *Col4A1*, *Col4A2*, *ITGA6*, *LAMA4*, *LAMB1*, *LAMC1*, *VWF*, *ITGA5*, *THBS1*, *PDGFB*, *PGF*, *RHOC*, and *CTNNB1*. Cell metabolism-relevant genes, e.g., *MGST3*, *PNP*, *UPP1*, and *HYAL2* might be the prospective therapeutic targets. Furthermore, we found that DEGs only in the upper endothelial cells, such as *MAPK3*, *STAT3*, *RHOA*, *MAPK11*, *HIF1A*, *FGFR1*, *GNG5*, *GNB1,* and *ARHGEF12*, mainly regulated cell adhesion, structure morphogenesis, and motility through Phospholipase D, Apelin, and VEGF signaling pathways. Moreover, DEGs only in the lower endothelial cells, for instance *PLCG2*, *EFNA1*, *CALM1*, and *RALA*, mainly regulated cell apoptosis and survival by targeting calcium ion transport through Rap1, Ras, cAMP, Phospholipase D, and Phosphatidylinositol signaling pathways. In addition, the upper endothelial cells showed significant functional diversity such as cytokine-responsive, migratory, and proliferative capacity, presenting a better angiogenic capacity and making it more sensitive to anti-angiogenic therapy compared with the lower endothelial cells. Our study has identified the potential targeted genes for anti-angiogenic therapy for both upper and lower ESCC, and further indicated that anti-angiogenic therapy might be more effective for upper ESCC, which still need to be further examined in the future.

## 1. Instruction

Esophageal carcinoma is the seventh most common cancer type worldwide with 604,000 new cases and 544,000 deaths annually [1]. The incidence of esophageal cancer significantly differs between the two most common histologic subtypes, esophageal squamous cell carcinoma (ESCC) and esophageal adenocarcinoma (EAC) [1,2]. ESCC incidence has declined in China recently but still causes approximately half of the world’s new ESCC cases [1,3]. Cancer epidemiology statistics have reported that alcohol intake, tobacco smoking, micronutrient deficiency, and dietary carcinogen exposure will cause ESCC [4,5,6], and additional suspected risk factors such as betel quid chewing and consumption of pickled vegetables and very hot food and beverages are also serious and regionally dependent [1].

ESCC is a highly aggressive disease with poor prognosis due to the lack of targeted therapies [7,8], inherent resistance to chemotherapy drugs [9], and locally advanced or distant metastasis [10]. As reported previously, PD-1 antibodies [11], pembrolizumab, and nivolumab [12] were applied in clinical treatment for patients with advanced ESCC for whom first-line chemotherapy failed, whereas all of them only moderately ameliorated the effect on the overall survival compared with chemotherapy [11,12]. Therefore, it is urgent to explore novel therapeutic targets for ESCC.

Considering that ESCC development and metastasis are closely related to the level of tissue vascularization [13,14,15,16], exploring anti-angiogenic targets is promising and critical for ESCC treatment. Previously, to find new therapeutic or prognosis targets, RNA sequencing was performed to explore the genomic changes and key regulatory factors on cell metastasis, epithelial-mesenchymal transition (EMT), and angiogenesis [17]. However, cellular heterogeneity in ESCC should be taken into account; therefore, researchers began to identify cell subtypes of ESCC and explore the genomic landscape of each subset separately to obtain more precise therapeutic targets through single cell RNA sequencing (scRNA-seq) [18,19]. Some researchers even detected genomic alterations in ESCC patients with alcohol-drinking through scRNA-seq and found *LRP1B*, *TTC28*, aberrant cell cycle, and PI3K-AKT pathways were critical in ESCC, revealing the potential therapeutic targets and gene mutation signature caused by drinking alcohol [20].

Clinically, ESCC can occur in the upper, middle, and lower esophagus [21]. Moreover, ESCC locating on the upper and the lower esophagus always showed different exhibition after surgery or chemotherapy in a clinic. For example, upper ESCC is more sensitive to chemotherapy than lower ESCC, whereas lower ESCC always shows a better prognosis than upper ESCC after surgical treatment, but the underlying mechanism is not clear [22]. Therefore, ESCC location should be taken into account when exploring new therapeutic targets through scRNA-seq.

In the current study, we harvested two upper ESCC and two lower ESCC and utilized scRNA-seq to identify endothelial cells in upper and lower ESCC. Compared with other cell subtypes in upper and lower ESCC, differently expressed genes (DEGs) in endothelial cells (the upper endothelial cells and the lower endothelial cells) were identified and common expressed DEGs in the upper and the lower endothelial cells, and DEGs only existing in the upper or the lower endothelial cells were screened out. Subsequently, gene ontology (GO) and Kyoto Encyclopedia of Genes and Genomes (KEGG) analysis were further performed to find prospective novel therapeutic targets.

## 2. Methods and Materials

### 2.1. Sample Collection and Preparation

This study included four patients (male; age 49–64; BMI 18.7–23.0)) with pathological diagnosis of ESCC (2 upper and 2 lower ESCC) for scRNA-seq analysis. All the experimental procedures were carried out following the ethical principles and protocols approved by First Hospital of Quanzhou Affiliated to Fujian Medical University (Quanzhou, China) and Huaqiao University (Quanzhou, China). Patient consent was obtained before the surgery. All patients did not receive chemotherapy, radiotherapy, or any other antitumor drug treatment before tumor resection. After surgical resection, the upper and lower esophagus tissues of ESCC were obtained, respectively.

### 2.2. Single Cell RNA Sequencing and Sequencing Library Preparation

Database building used Cell Ranger single cell software suite (Version 4.0, 10xgenomics, San Francisco, CA, USA). The sample was first prepared into a single-cell suspension, and then the cell count and cell viability were detected. In cell sorting using microfluidic system, gel beads with barcode enter at a uniform speed from the left, cells and enzymes to be sorted enter from below at a certain time interval and combine with gel beads to enter the oil phase to form GEMs (Gel Bead in emulsion) [23]. Subsequently, the cells ruptured, and under the action of reverse transcriptase, mRNA was reverse transcribed into cDNA. After PCR amplification, a sequencing library was constructed and placed on a sequencer for sequencing. We performed double-end sequencing of scRNA-seq data on the Illumina sequencing platform and compared it with the reference genome of GRCh38 to generate the original gene expression matrix of each sample. Sequencing was by Guangzhou Genedenovo Biotechnology Co., Ltd, Guangzhou, China.

### 2.3. Preprocessing of scRNA-Seq Data

The raw matrices were imported into the Seurat (version 4.1.1, Satija Lab, New York, NY, USA) R toolkit for quality control and downstream analysis. For quality screening, we filtered cells with greater than 5000 or less than 200 unique feature counts, filtering cells with greater than 25% mitochondrial counts. The global scaling normalization method (LogNormalize) was used to normalize the eigenvalues of each cell and multiply them by a scaling factor (generally 10,000). Finally, the results were logarithmically converted. After quality standardization, the Seurat R package was applied to analyze the scRNA-seq data. Using the ‘FindVariableGenes’ function, highly variable genes were selected according to the average expression level and the scatter level threshold [24]. By default, 2000 highly variable genes were returned for downstream analysis. The ‘ScaleData’ function was normalized to change and scale the expression of each gene, and principal component analysis (PCA) was performed by RunPCA. According to ‘ElbowPlot’, we determined the number of principal components (PCs).

### 2.4. Clustering Analysis and Nonlinear Dimensionality Reduction

Since the data of the upper and lower organizations were fused by four sample data, in order to reduce the difference between batches the data of multiple batches were recombined as much as possible. Therefore, harmony (version 0.1.0, Ilya Korsunsky Massachusetts, USA) [25] R toolkit was used for batch effect processing, and the resolution parameter (Res) was set to 0.3. We used ‘FindClusters’ to cluster the first 32 principal components of ESCC upper tissue. The same treatment was performed on the tissue of the lower segment of ESCC, in which 42 principal component analyses (PCA) were performed, and other parameters were the same as those of the upper segment. Cluster-specific marker genes were determined using the ‘FindAllMarkers’ function. Difference multiple and expression ratio parameter settings were set to 0.25 (min.pct = 0.25, logfc.threshold = 0.25). According to the marker genes and the CellMarker dataset, we annotated different cell types (cell clusters were annotated with the dominant expression cell markers). Finally, gene expression and clustering results were displayed on the tSNE plot using ‘RunTSNE’. Subpopulations were defined by DEGs and biological background.

### 2.5. Identification and Cell Ratio of Endothelial Cells in the Upper and Lower ESCC

Endothelial cells were marked using *VWF, PLVAP*, *PECAM1*, *CD34*, *TIE1*, and *CDH5* and visualized through violin plots and coordinate maps to locate the subpopulation of endothelial cells. After subset identification in the upper and lower ESCC, cell numbers of endothelial cells and other subsets were counted, respectively. We used the ‘percent’ function in the scales (version, 1.2.0) R toolkit to calculate the ratio of endothelial cells in the total cell population and drew a pie chart using the ‘pie’ function.

### 2.6. GO and KEGG Enrichment Analysis

Compared with other cell subtypes in upper and lower ESCC, DEGs in the upper and the lower endothelial cells were obtained by filtering by setting the parameters ‘only.pos = TRUE, min.pct = 0.25, logfc.threshold = 0.25’ using the ‘FindAllMarkers’ function in Seurat. This function is used to compare the gene expression between a cluster and all other clusters. The selected genes only show the genes with positive expression in the current cluster. Highly expressed genes are helpful for cluster annotation and later enrichment analysis. Significantly expressed genes were screened out again by UMI count. Among them, the genes in the endothelial cells of the upper and lower tissue were screened. Venn diagram was used to screen common genes shared by the upper and lower ESCC, genes only in upper ESCC, and genes only in lower ESCC. GO and KEGG analyses were performed utilizing Omicshare online software [26], and genome enrichment analysis was performed by mapping all significantly expressed genes to terms in the KEGG database and the GO database. GO analysis can be divided into three parts, namely biological process (BP), cellular component (CC), and molecular function (MF). Through KEGG analysis, we can learn in which pathways genes are involved and the annotations of their own functions.

### 2.7. Screening of Targeted Genes

DEGs involved in the top 5 enriched pathways obtained through KEGG assay were collected, and the common DEGs shared by these pathways were screened using Venn diagram. Finally, DEGs involved in 5, 4, and at least 3 pathways were collected and considered as potential therapeutic targets.

### 2.8. Clustering Analysis of the Endothelial Cells and Nonlinear Dimensionality Reduction

Clustering analysis of the endothelial cells in upper and lower ESCC was furtherly performed according to Section 2.4. The resolution parameter (Res) was set to 0.9, using ‘FindClusters’ to cluster the first 14 principal components. The detailed analysis method is consistent with that used in 2.4. Subsequently, cell features were analyzed and identified.

### 2.9. Single-Cell Trajectory Analysis

We applied Monocle (version, 2.22.0, Cole Trapnell, Seattle, WA, USA) on the identified endothelial cells to predict cell differentiation as well as developmental trajectories. Without special instructions, the function uses default parameters. First, highly variable genes (HVG) were selected using the ‘VariableFeatures’ function in Seurat v4. Then, we used the ‘reduceDimension’ and ‘orderCells’ functions for dimension reduction and sorting. Data reduction used the Reverse Graph Embedding (DDRTree) Algorithm. Then, color treatment was performed according to the cell type. Finally, we obtained the trajectory map and defined the starting point and end point of the map according to the research background.

## 3. Results

### 3.1. Endothelial Cells Isolated from Upper ESCC through scRNA Sequencing Analysis

**Figure 1:**
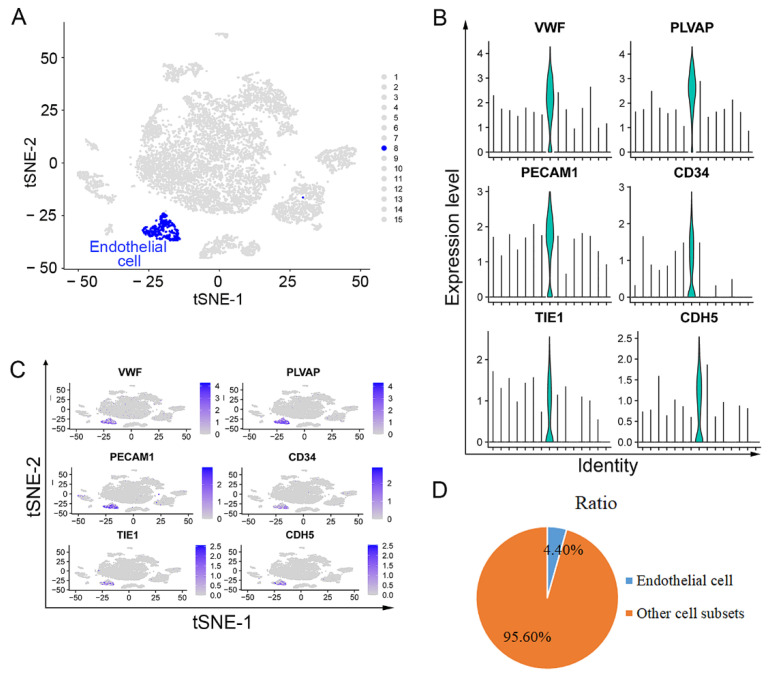
Gene profile of endothelial cells in human upper ESCC with scRNA sequencing: (**A**) tSNE map of total 9891 high-quality cells and the cluster visualization of endothelial cells; blue dots: endothelial cells. (**B**) expression levels of *VWF*, *PLVAP*, *PECAM1*, *CD34*, *TIE1*, and *CDH5* in 9891 cells illustrated as Violin plots; and (**C**) tSNE map; (**D**) ratio of endothelial cells and other cell subsets.

To visualize endothelial cells in upper ESCC, we detected single-cell gene expression of two upper fresh, surgically removed ESCC tumors. After scRNA-seq and PCA analysis, the upper ESCC tissue was divided into 15 subgroups (Figure 1A). Endothelial cells were annotated using the specific markers (Figure 1A). The distributions and expression levels of these classic markers (*VWF*, *PLVAP*, *PECAM1*, *CD34*, *TIE1*, and *CDH5*) in endothelial cells were consistent with the annotation (Figure 1B,C). All these findings suggested that the eighth subgroup was endothelial cells. The ratio of endothelial cells in total cell population was about 4.40%, and the ratio of other cell subsets was 95.6% (Figure 1B).

### 3.2. Endothelial Cells Isolated from Lower ESCC through scRNA Sequencing Analysis

**Figure 2:**
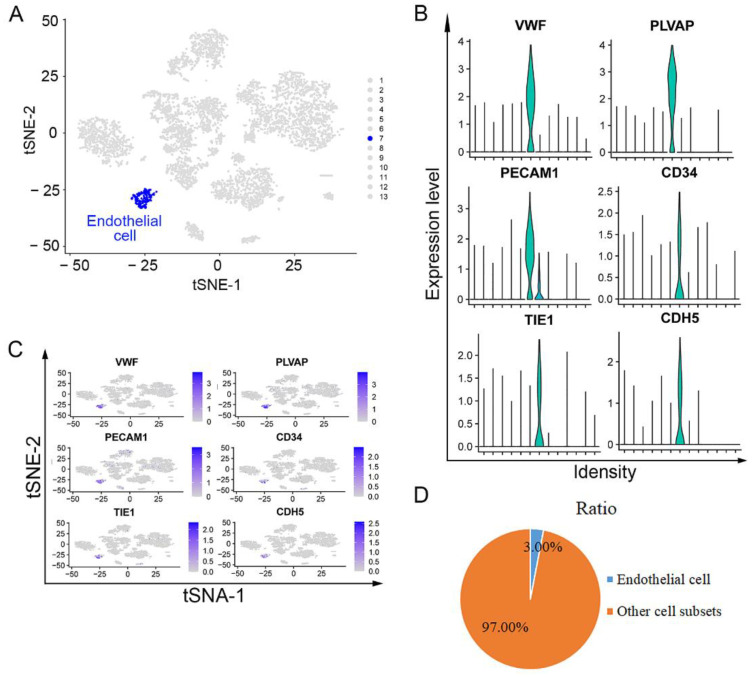
Gene profile of endothelial cells in human lower ESCC with scRNA sequencing: (**A**) tSNE map of total 6262 high-quality cells and the cluster visualization of endothelial cells; blue dots: endothelial cells. (**B**) expression levels of *VWF*, *PLVAP*, *PECAM1*, *CD34*, *TIE1*, and *CDH5* in 6262 cells illustrated as Violin plots; and (**C**) tSNE map; (**D**) ratio of endothelial cells and other cell subsets.

Endothelial cells in lower ESCC were also verified using scRNA-seq and PCA analysis of two lower fresh, surgically removed ESCC tumors. The lower tissue was divided into 13 subgroups (Figure 2A). Endothelial cells were annotated using the same markers as mentioned above (Figure 2A). The expression levels and distributions of these classic markers (*VWF*, *PLVAP*, *PECAM1*, *CD34*, *TIE1*, and *CDH5*) in endothelial cells were consistent with the annotation (Figure 2B,C). The ratio of endothelial cells in total cell population was about 3.00%, and the ratio of the other cell subsets was 97.0% (Figure 2D). Similarly, these findings indicated that the seventh subset was the endothelial cells.

### 3.3. Detection, GO, and KEGG Enrichment Analysis of Common DEGs

**Figure 3:**
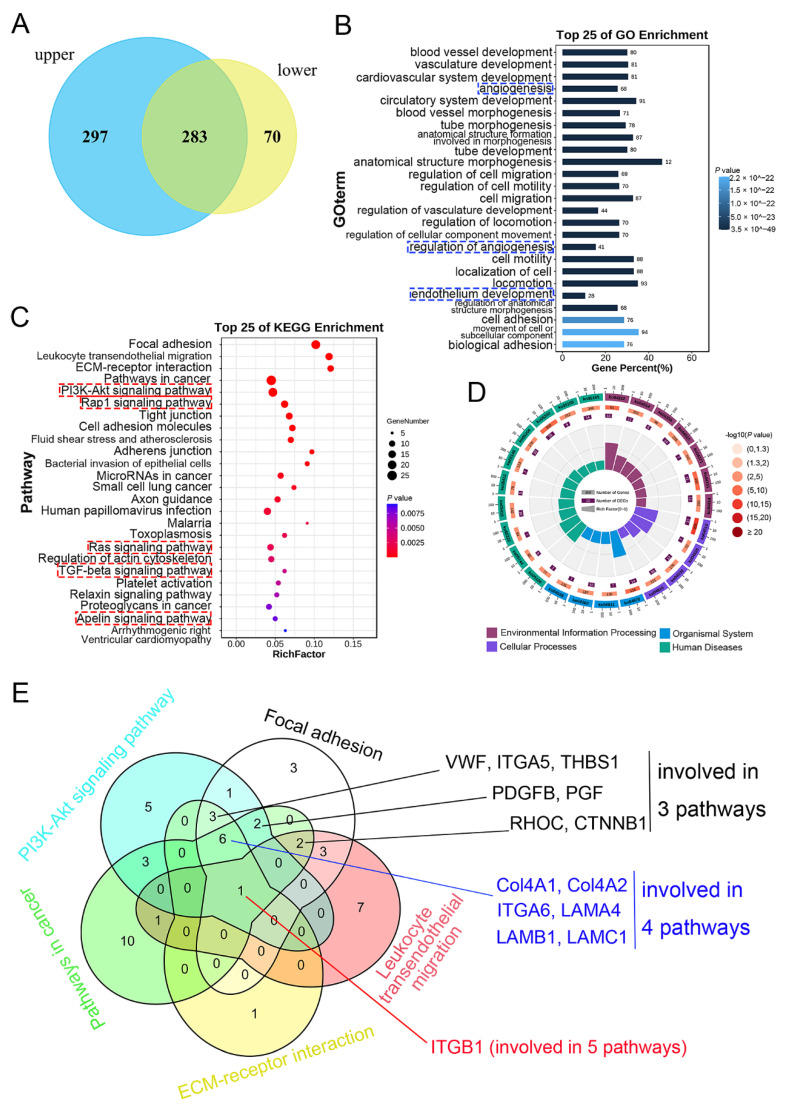
GO and KEGG enrichment analysis of the common DEGs: (**A**) common expressed DEGs, DEGs only in the upper or lower endothelial cells were presented using Venn diagram; (**B**) GO function enrichment; (**C**) KEGG pathway enrichment was shown in gradient; and (**D**) circular diagram; (**E**) common DEGs involved in the top 5 pathways. Blue box: the three functions with the highest rich factors; red box: the critical five pathways with the highest rich factors.

Compared with other cell subtypes in upper and lower ESCC, the DEGs in the upper and the lower endothelial cells were analysed, and the genes with *p* value < 0.01 and log2 (FC) > 0.25 were considered as significant. Finally, 580 DEGs in the upper endothelial cells and 353 DEGs in the lower endothelial cells were obtained (Figure 3A). Subsequently, common DEGs existing in both the upper and the lower endothelial cells were presented in Venn diagram. As shown in Figure 3A, 283 genes were found to be common DEGs shared by the upper and the lower endothelial cells, and 297 DEGs existed in the upper endothelial cells but only 70 DEGs in the lower endothelial cells. Furthermore, the GO analysis showed that the biological processes of the common genes were enriched in vessel development, angiogenesis, and cell motility of endothelial cells (Figure 3B). The three functions with the highest rich factor were angiogenesis, regulation of angiogenesis, and endothelium development (Figure 3B). Moreover, KEGG was furtherly carried out and found that these common DEGs might mainly regulate environmental information processing, organism systems, cellular processes, and human diseases through PI3K-AKT, Rap1, Ras, TGF-beta, Apelin signaling pathways, and so on (Figure 3C,D). DEGs shared in the top five pathways were detected using Venn diagram to screen the potential targeted genes for treatment, and the genes involved in at least three pathways were collected. Finally, *ITGB1* (involved in five pathways), *Col4A1*, *Col4A2*, *ITGA6*, *LAMA4*, *LAMB1*, and *LAMC1* (involved in four pathways), *VWF*, *ITGA5*, *THBS1*, *PDGFB*, *PGF*, *RHOC*, and *CTNNB1* (involved in three pathways) were obtained (Figure 3E). Additionally, DEGs involved in cell metabolism were also the prospective therapeutic targets, and *UPP1*, *MGST3*, *MGST2*, *AK1*, *PNP*, *DAD1*, *HYAL2*, *MGLL*, *COX7A1*, and *PGM2L1* were obtained (Figure S1).

### 3.4. Optimization of the Targeted Genes

**Figure 4:**
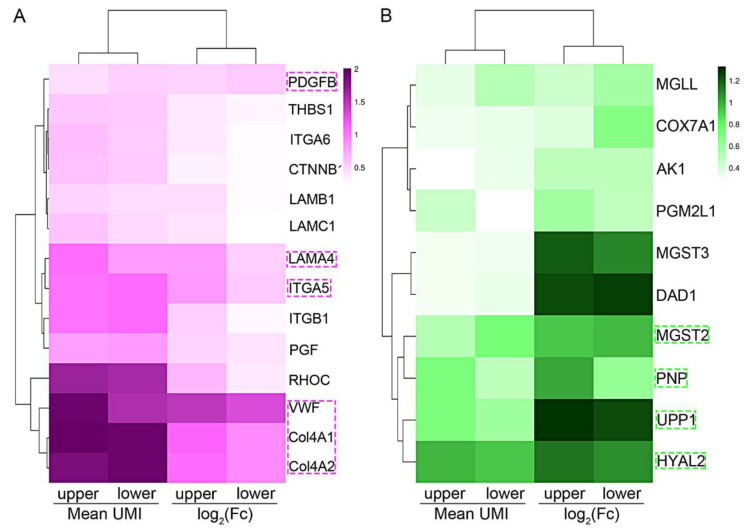
Heatmap of the mean UMI and log2(FC) of the common genes screened from the top five KEGG pathways and metabolism: (**A**) heatmap of the genes in KEGG pathways; and (**B**) cell metabolism. Purple box: the genes with mean UMI > 0.5 and log2(FC) > 0.5; green box: the genes with mean UMI > 0.5 and log2(FC) > 0.5.

Common DEGs existed in both the upper and the lower endothelial cells and involved in the top five KEGG pathways, and cell metabolism was collected. Since ideal targeted genes for treatment should have a high expression level and good specificity, we further screened the DEGs with the standard of mean UMI > 0.5 and log2(FC) > 0.5. As shown in Figure 4, only *PDGFB, LAMA4, ITGA5, VWF, Col4A1,* and *Col4A2* (involved in the top five pathways), *MGST2*, *PNP*, *UPP1*, and *HYAL2* (involved in cell metabolism) were harvested. In addition, the remaining DEGs might not be suitable for both the upper and the lower endothelial cell treatment, whereas some DEGs could be applied for treatment targeting the upper endothelial cells only, e.g., *ITGB1*, *RHOC*, and *MGLL* for the lower endothelial cells (Figure 4).

### 3.5. GO and KEGG Enrichment Analysis of DEGs Only Expressed in Upper Endothelial Cells

**Figure 5:**
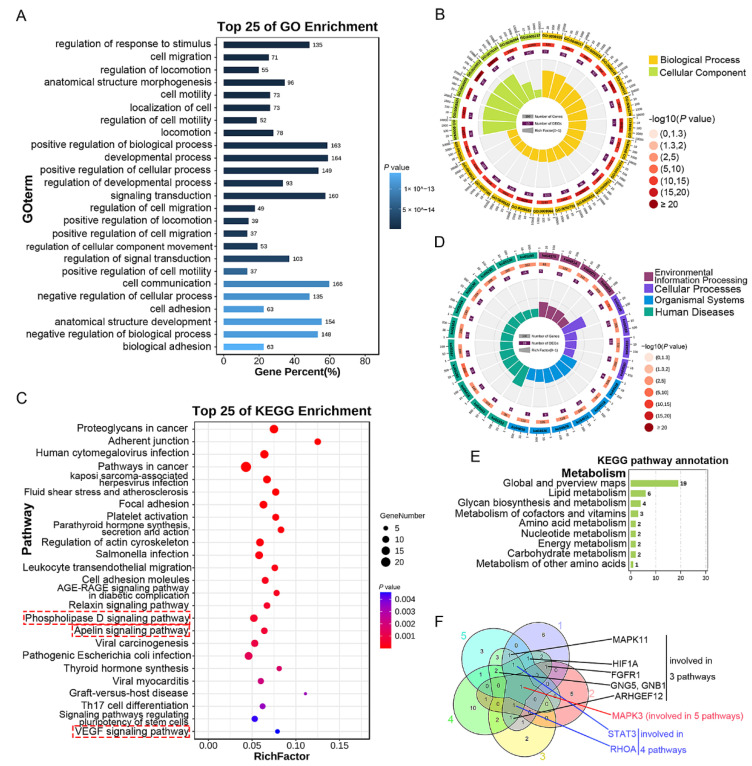
GO and KEGG enrichment analysis of the DEGs only expressed in the upper endothelial cells: (**A**) GO function enrichment of the DEGs only in the upper endothelial cells gradient; and (**B**) circular diagram; (**C**) KEGG pathway enrichment was showed in gradient; and (**D**) circular diagram; (**E**) KEGG pathways annotation in metabolism; (**F**) common DEGs involved in the top five pathways.

The GO analysis showed that the biological processes of the DEGs only in the upper endothelial cells were enriched in cell adhesion, cell locomotion, and cell motility of endothelial cells (Figure 5A,B). In addition, cellular components encoded by these DEGs mainly regulated the focal junction, the cell-substrate junction, and the adheres junction (Figure 5B and Figure S2). KEGG was also performed and found that these DEGs in the upper endothelial cells might mainly regulate environmental information processing, cellular processes, organism systems, and human diseases through Phospholipase D, Apelin, and VEGF signaling pathways (Figure 5C,D). Cell metabolism regulated by these pathways and relevant genes are shown in Figure 5E and Table 1. All the DEGs simultaneously involved in the top five pathways were furtherly detected using Venn diagram to screen the potential targeted genes for treatment with upper ESCC, and the genes involved in at least three pathways were collected. Finally, *MAPK3* (involved in five pathways), *STAT3* and *RHOA* (involved in four pathways), *MAPK11, HIF1A, FGFR1, GNG5, GNB1*, and *ARHGEF12* (involved in three pathways) were obtained (Figure 5F).

### 3.6. GO and KEGG Enrichment Analysis of DEGs Only Expressed in Lower Endothelial Cells

**Figure 6:**
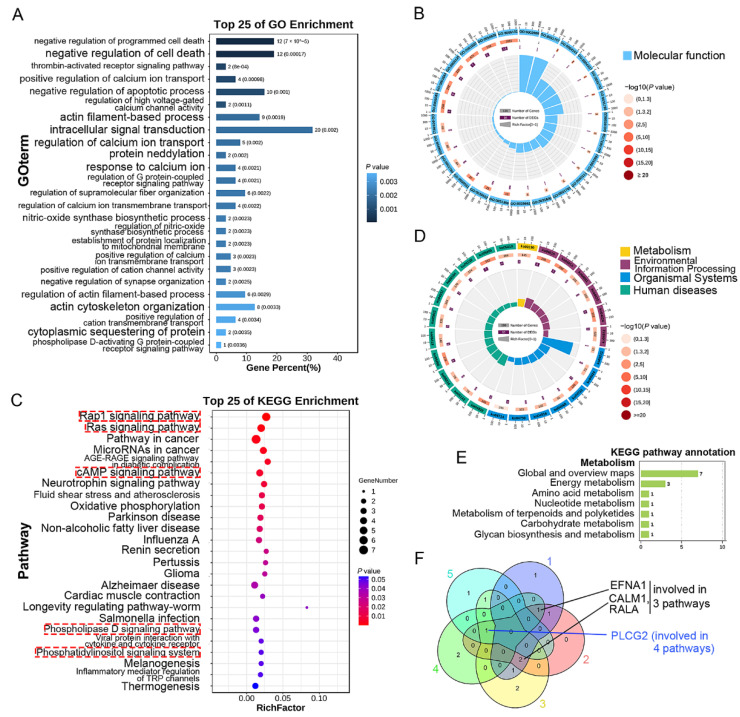
GO and KEGG enrichment analysis of the DEGs only expressed in the lower endothelial cells; (**A**) GO function enrichment of the DEGs only in the lower endothelial cells gradient; and (**B**) circular diagram; (**C**) KEGG pathway enrichment was showed in gradient; and (**D**) circular diagram; (**E**) KEGG pathways annotation in metabolism; (**F**) common DEGs involved in the top 5 pathways.

Functional enrichment analysis of the lower endothelial cells was performed consistent with the upper endothelial cells. The biological processes of the DEGs only in the lower endothelial cells were enriched in cell survival, calcium ion transport, and cytoskeleton organization of endothelial cells (Figure 6A,B). In addition, function was mainly enriched in molecular function, such as DNA methylation and demethylation, endothelin A receptor binding, and endothelial differentiation (Figure 6B and Figure S3). KEGG was also carried out and found that these DEGs in the lower endothelial cells might mainly regulate cell metabolism, environmental information processing, organism systems, and human diseases through Rap1, Ras, cAMP, Phospholipase D, hosphatidylinositol signaling pathways, and so on (Figure 6C,D). Cell metabolism regulated by these pathways and relevant genes is shown in Figure 6E and Table 2. All the DEGs simultaneously involved in the top five pathways were also further detected using Venn diagram to screen the potential targeted genes for anti-angiogenic treatment with lower ESCC. Similarly, the genes involved in at least three pathways were collected. Finally, *PLCG2* (involved in four pathways), *EFNA1, CALM1,* and *RALA* (involved in three pathways) were obtained (Figure 6F).

### 3.7. Clustering Analysis of the Upper and Lower Endothelial Cells

**Figure 7:**
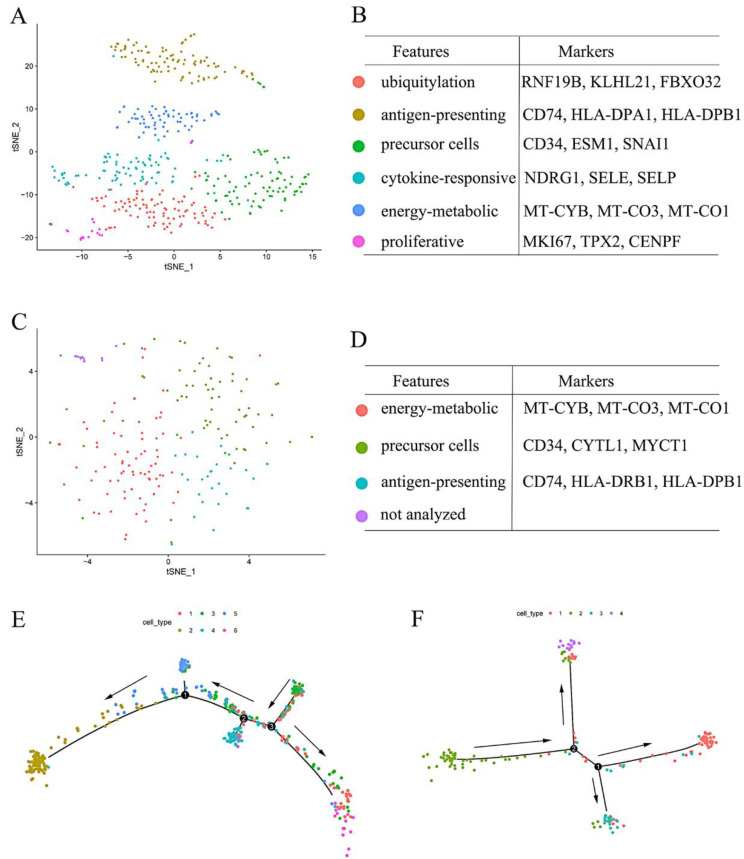
Clustering analysis of the upper and the lower endothelial cells: tSNE map, cluster visualization and its gene markers of the (**A,B**) upper; and (**C,D**) the lower endothelial cells. Trajectory analysis of the subsets in (**E**) the upper; and (**F**) the lower endothelial cells. Black arrow: development direction.

To verify more detailed differences, we performed PCA on the upper and lower endothelial cells and further analyzed interactions among these subsets utilizing trajectory analysis. In the upper endothelial cells, we finally obtained six subsets with different functions and markers: ubiquitylation cells (*RNF19B, KLHL21, FBXO32*), antigen-presenting cells (*CD74, HLA-DPA1, HLA-DPB1*), precursor cells (*CD34, ESM1, SNAI1*), cytokine-responsive cells (*NDRG1, SELE, SELP*), energy-metabolic cells (*MT-CYB, MT-CO3, MT-CO1*), and proliferative cells (*MKI67, TPX2, CENPF*) (Figure 7A,B). However, only four subsets were found in the lower endothelial cells and identified using gene markers: energy-metabolic cells (*MT-CYB, MT-CO3, MT-CO1*), precursor cells (*CD34, CYTL1, MYCT1*), antigen-presenting cells (*CD74, HLA-DRB1, HLA-DPB1*), and the fourth subset (Figure 7C,D). Although subgroup four expressed some special genes, such as anti-apoptotic genes and exosome markers, cell number and ratio were too small and made it unrepresentative (Figure 7C,D); therefore, we did not identify this subset further. Differentiation and developmental interactions of subsets of the upper endothelial cells are shown in Figure 7E. Endothelial precursor cells differentiated into ubiquitylation cells, cytokine-responsive cells, energy-metabolic cells, proliferative cells, and antigen-presenting cells, successively (Figure 7E). For the subsets in the lower endothelial cells, endothelial precursor cells differentiated into energy-metabolic cells and antigen-presenting cells, whereas they showed significant overlap in the cell developmental process (Figure 7F).

## 4. Discussion

Anti-angiogenesis therapy is confirmed to be an effective approach to inhibiting tumor development and metastasis through triggering ‘tumor starvation’ [27]. Endostatin and bevacizumab have already been applied in advanced ESCC treatment as anti-angiogenesis drugs through inhibiting VEGF cascade, cell proliferation of endothelial cells, and vessel development [28,29]. Nevertheless, high incidence of gene mutation and strong adaptability of tumor cells make ESCC susceptible to drug resistance; therefore, it is urgent to explore more novel therapeutic targets. In the current study, *ITGB1, Col4A1, Col4A2, ITGA6, LAMA4, LAMB1*, *LAMC1, VWF, ITGA5, THBS1, PDGFB, PGF, RHOC, CTNNB1* (involved in signaling transduction) and *UPP1, MGST3, MGST2, AK1, PNP, DAD1, HYAL2, MGLL, COX7A1*, and *PGM2L1* (involved in cell metabolism) were obtained and considered to be the potential anti-angiogenic targets for both upper and lower ESCC. Expression of the abovementioned genes in upper and lower ESCC showed significant differences compared with that in endothelial cells in normal esophageal tissue (Figures S4 and S5), further verifying the reliability of the above conclusions. Furthermore, *MAPK3, STAT3, RHOA, MAPK11, HIF1A, FGFR1, GNG5, GNB1,* and *ARHGEF12* were identified as the critical regulatory genes in the upper endothelial cells only, whereas PLCG2, EFNA1, CALM1, and RALA were screened out in the lower endothelial cells. In addition to energy-metabolic cells, antigen-presenting cells, and precursor cells, the upper endothelial cells had more obvious cell heterogeneity, e.g., ubiquitylation cells, cytokine-responsive cells, and proliferative cells compared with the lower endothelial cells.

During the process of tumor development, to stimulate the growth of blood vessels and extend to tumor tissue, most tumor cells will synthesize and secrete the matrix metalloproteinase (MMPs) [30]. Subsequently, MMPs accelerate extracellular matrix degradation and further supply a suitable approach for (precursor) endothelial cell migration [31]. Then, (precursor) endothelial cells migrate towards tumor tissue along the approach through regulating the cytoskeleton structure by ubiquitylation cascade [32,33]. After close to the vessel budding sites, (precursor) endothelial cells will respond to the proangiogenic signals and begin to proliferate to form new capillary blood vessels towards the tumor tissue and supply nutrition, oxygen, and routes for ESCC growth and metastasis, such as VEGF, platelet-derived growth factor (PDGF), and fibroblast growth factor (FGF) [30,34], thereby blocking these signaling pathways and attenuating ESCC progression. Endothelial cells in the upper ESCC can be divided into different subsets covering the whole angiogenic process, such as precursor, ubiquitylation, cytokine-responsive, and proliferative endothelial cells (Figure 7E), showing a more activated status. Rapidly proliferating or mitotic cells will be more sensitive to chemotherapy drugs; therefore, chemotherapy or anti-angiogenic therapy might be more effective in upper ESCC than in lower ESCC.

The abovementioned angiogenic processes are the major anti-angiogenic targets, and some critical genes were confirmed to be effective previously and consistent with our findings. STAT3 is a key transcription factor involved in wide cellular processes, and a previous study found blocking the STAT3 pathway was of benefit to inhibit angiogenesis and ESCC growth [35,36]. However, our findings indicated that blocking STAT3 might only be applicable to upper ESCC. Similarly, RHOC was also proved to be the cancer therapeutic target due to its critical regulatory effects on cytoskeleton organization of cancer stem cells [37]. Our findings also confirmed RHOC was a potential therapeutic target for both upper and lower ESCC. Whether the remaining genes obtained in the current study are suitable for anti-angiogenesis needs further exploration.

We believe that position effects cannot be ignored when exploring potential therapeutic targets for tumors with the same histological characterization but located in different positions. Ideal therapeutic targets for cancer treatment should be specific with high expression in the targeted cells and key molecules involved in cell proliferation, migration, survival, differentiation, and so on. Therefore, these targeted genes should be optimized according to the standards.

## 5. Conclusions

In the current study, potential targeted genes for anti-angiogenesis in both upper and lower ESCC treatment were preliminarily screened out, including ITGB1, Col4A1, Col4A2, ITGA6, LAMA4, LAMB1, and LAMC1, VWF, ITGA5, THBS1, PDGFB, PGF, RHOC, and CTNNB1 (involved in signaling transduction) and UPP1, MGST3, MGST2, AK1, PNP, DAD1, HYAL2, MGLL, COX7A1, and PGM2L1 (involved in cell metabolism). Moreover, MAPK3, STAT3, RHOA, MAPK11, HIF1A, FGFR1, GNG5, GNB1, and ARHGEF12 were the potential therapeutic targets for upper ESCC only, whereas PLCG2, EFNA1, CALM1, and RALA were only suitable for lower ESCC. Based on the results, we predict that upper ESCC may be more sensitive to chemotherapy.

## Figures and Tables

**Table 1:** DEGs involved in regulation of substance metabolism in the upper endothelial cells.

Metabolism	Genes
Lipid metabolism	*PLPP1*, *PLPP3*, *PLA2G16*, *SPHK1*, *DGKZ*, *GPX1*
Glycan biosynthesis and metabolism	*GNS*, *CSGALNACT1*, *NDST1*, *GALNT18*
Metabolism of cofactors and vitamin	*HMOX1*, *NNMT*, *NT5E*
Amino acid metabolism	*SAT1*, *KMT2E*
Nucleotide metabolism	*NT5E*, *GUK1*
Energy metabolism	*MT-ATP6*, *MT-ND5*
Carbohydrate metabolism	*CYB5R3*, *PLCB1*
Metabolism of other amino acid	*GPX1*

**Table 2:** DEGs involved in regulation of substance metabolism in the lower endothelial cells.

Metabolism	Genes
Energy metabolism	*UQCR11*, *NDUFB2*, *COX7A2*
Amino acid metabolism	*COLGALT1*
Nucleotide metabolism	*PDE4B*
Metabolism of terpenoids and polyketides	*FDPS*
Carbohydrate metabolism	*PLCG2*
Glycan biosynthesis and metabolism	*COLGALT1*

## Data Availability

The data presented in this study are available on request from the corresponding author.

## Supplementary Materials

The following supporting information can be downloaded at: https://www.mdpi.com/article/10.3390/curroncol29100607/s1. Figure S1: Common DEGs involved in regulation of substance metabolism in both upper and lower endothelial cells. Figure S2: Cellular component enrichment analysis of the DEGs in upper endothelial cells. Figure S3: Molecular function enrichment analysis of the DEGs in lower endothelial cells. Figure S4: Heatmap of the screened common genes involved in KEGG pathways. Figure S5: Heatmap of the screened common genes involved in metabolism.
