# Supplementary material for: Single-Cell Transcriptomics of Endothelial Cells in Upper and Lower Human Esophageal Squamous Cell Carcinoma
Source: Curr Oncol. 2022 Oct 14;29(10):7680–94. doi: 10.3390/curroncol29100607 (PMC9600084; doi:10.3390/curroncol29100607)
Supplement: Supplementary file 1 [file curroncol-29-00607-s001.zip › curroncol-1839943-supplementary.pdf]

Supplementary figures and tables

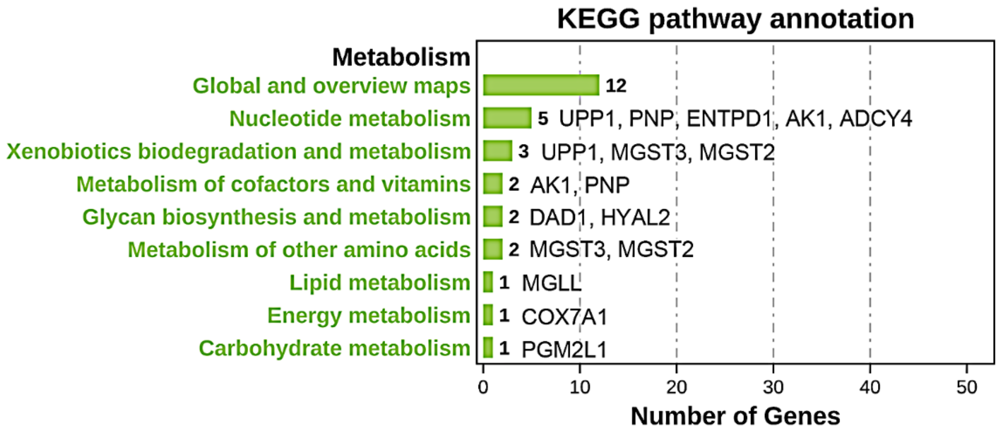

Figure S1

Common DEGs involved in regulation of substance metabolism in both upper and lower endothelial cells.

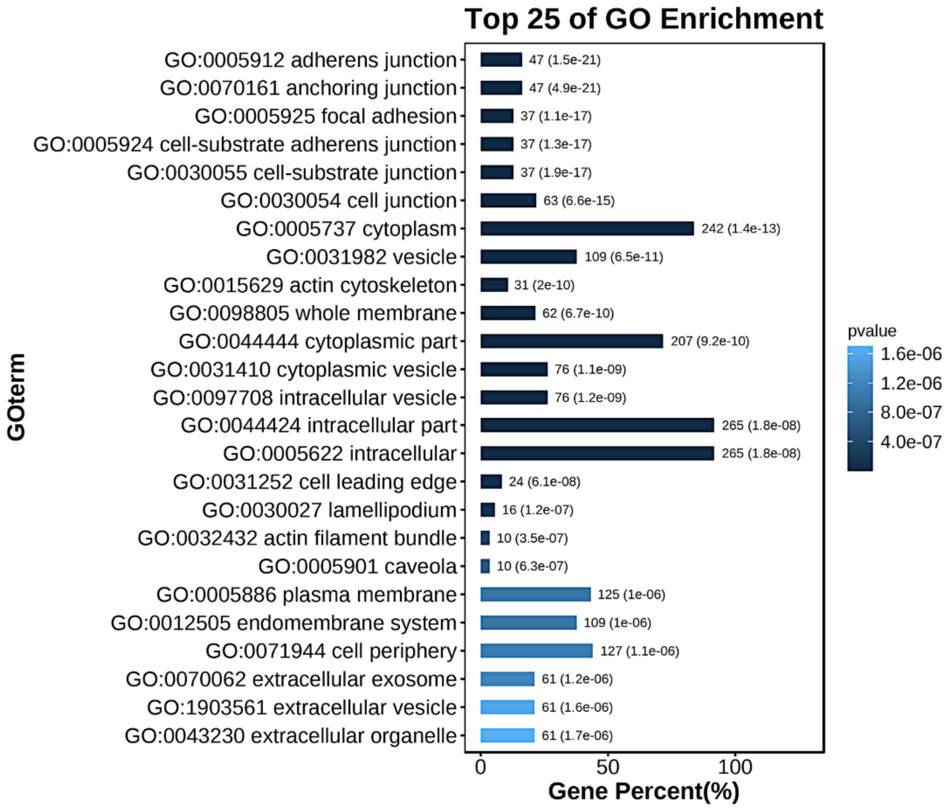

Figure S2

Cellular component enrichment analysis of the DEGs in upper endothelial cells.

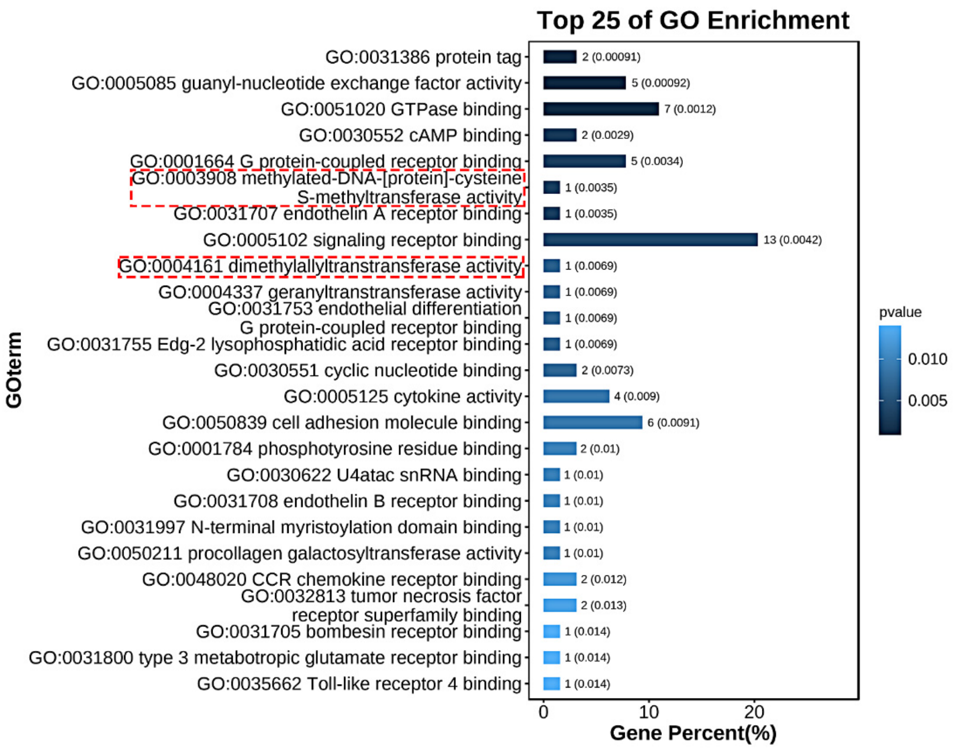

Figure S3

Molecular function enrichment analysis of the DEGs in lower endothelial cells.

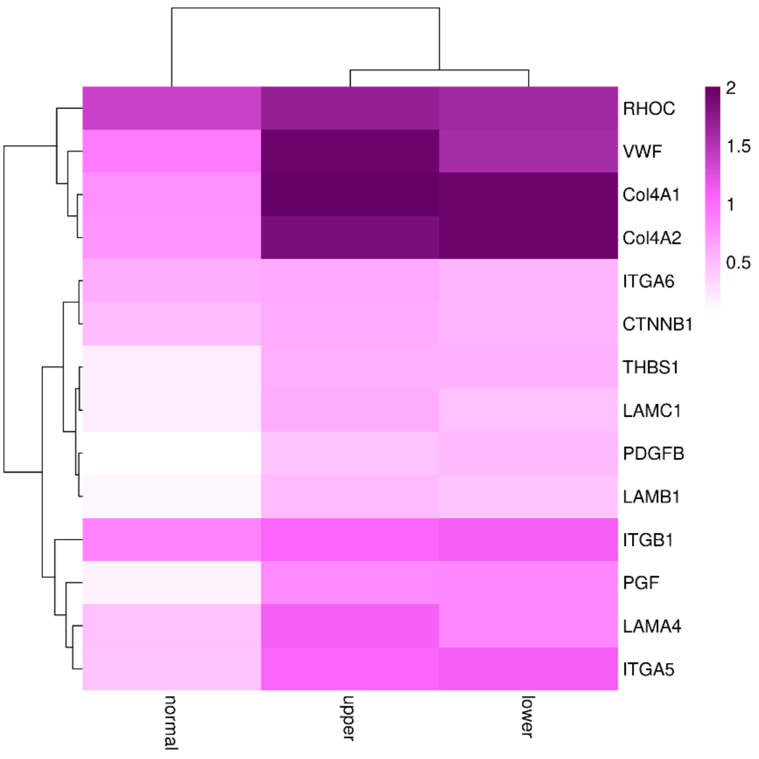

Figure S4

Heatmap of the screened common genes involved in KEGG pathways.

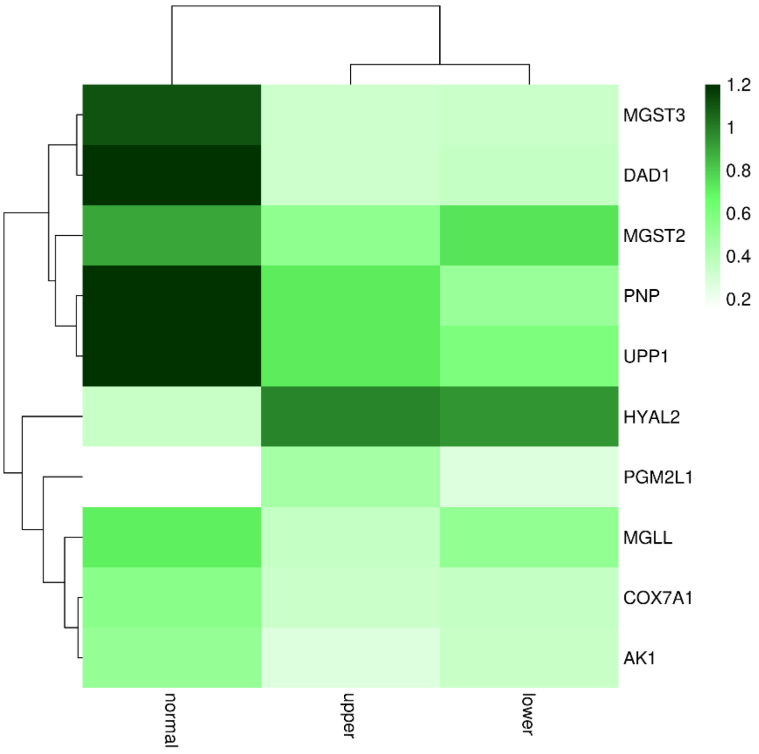

Figure S5

Heatmap of the screened common genes involved in metabolism.
